# A Phase 1 clinical trial to evaluate the safety and tolerability of CLZ-2002 for the treatment of patients with Charcot–Marie–Tooth disease type 1

**DOI:** 10.1093/stcltm/szag048

**Published:** 2026-07-28

**Authors:** Hyesun Kim, Byung-Ok Choi, Hyunju Lee, Saeyoung Park, Jaeseung Lim, Sung-Chul Jung

**Affiliations:** Cellatoz Therapeutics Inc., HIP of SNUH, 172, Dolma-ro, Bundang-gu , Seongnam-Si, Gyeonggi-do 13605, Republic of Korea; Department of Neurology, Samsung Medical Center, Sungkyunkwan University School of Medicine, Seoul 06351, Republic of Korea; Cellatoz Therapeutics Inc., HIP of SNUH, 172, Dolma-ro, Bundang-gu , Seongnam-Si, Gyeonggi-do 13605, Republic of Korea; Department of Biochemistry, College of Medicine, Ewha Womans University, Seoul 07804, Republic of Korea; Cellatoz Therapeutics Inc., HIP of SNUH, 172, Dolma-ro, Bundang-gu , Seongnam-Si, Gyeonggi-do 13605, Republic of Korea; Department of Biochemistry, College of Medicine, Ewha Womans University, Seoul 07804, Republic of Korea

**Keywords:** Schwann cell, Charcot–Marie–Tooth disease, CMT1, clinical trial, Phase 1, cellular therapy

## Abstract

**Background:**

Charcot–Marie–Tooth disease type 1 (CMT1) is a rare, genetically diverse condition and represents the most prevalent form of inherited peripheral neuropathy, characterized by Schwann cell dysfunction resulting in progressive demyelination and muscle wasting. CLZ-2002, an allogeneic Schwann cell-like product derived from human tonsillar mesenchymal stem cells, has been developed as a regenerative therapy and investigated in CMT1 patients.

**Methods:**

A Phase 1, open-label, dose-escalation clinical trial was performed in nine patients with genetically diagnosed CMT1 (five with CMT1A, four with CMT1B). Participants were allocated to three dosing cohorts: 6 million (G1), 12 million (G2), or 24 million cells (G3). CLZ-2002 was delivered as a single intramuscular injection into the lower limbs. The primary objective was to assess safety and tolerability. Exploratory endpoints included Charcot–Marie–Tooth Neuropathy Score version 2 (CMTNSv2), Overall Neuropathy Limitation Score-leg (ONLS-leg), Functional Disability Score (FDS), electrophysiology, MRI, and circulating biomarkers.

**Results:**

No drug-related adverse reactions, serious adverse events, or dose-limiting toxicities occurred. Four participants experienced a total of five grade 1-2 treatment-emergent adverse events. By Week 24, improvements relative to baseline were noted in CMTNSv2 and ONLS-leg. Biomarker levels of NCAM1 and GDF15 declined at Week 4 but returned toward baseline by Week 24, reflecting the observed clinical trends.

**Conclusions:**

A single intramuscular dose of CLZ-2002 of up to 24 million cells was safe and well-tolerated in CMT1 patients. Exploratory efficacy assessments suggested possible clinical benefit, warranting continued investigation of CLZ-2002 in larger, controlled study populations.

## Graphical abstract

**Graphical Abstract:**
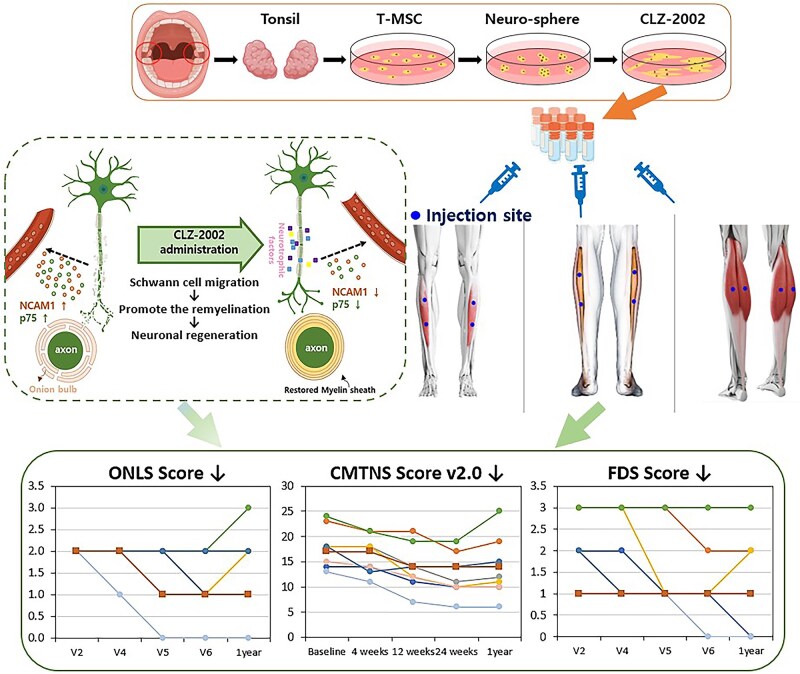

Significance statement Charcot–Marie–Tooth disease type 1 (CMT1) is an inherited neuropathy resulting from Schwann cell impairment. We generated CLZ-2002, allogeneic Schwann cell-like progenitors derived from human tonsillar mesenchymal stem cells, which underwent subsequent evaluation in a Phase 1 dose-escalation trial involving nine individuals with CMT1. Single intramuscular doses (6-24 million cells) were well tolerated, without serious adverse events or dose-limiting toxicity. Exploratory effectiveness measures, including CMTNSv2, ONLS-leg, and FDS, showed improvement by Week 24, while biomarker profiles for NCAM1 and GDF15 corresponded with clinical progression. CLZ-2002 demonstrated a favorable safety profile and potential therapeutic benefit, providing a rationale for further studies with expanded patient cohorts.

## Introduction

Charcot–Marie–Tooth (CMT) disease represents a group of inherited peripheral neuropathies that present as progressive motor and sensory impairment, resulting from mutations in various genes. Globally, CMT is estimated to affect around 1 in 2,500 people, making it one of the most prevalent inherited neurological disorders (though it is still considered to be a rare condition).^1–3^ In South Korea, the reported prevalence in 2018 was 5.2 per 100 000 population, affecting more men (6.1) than women (4.4).^4^^,^^5^ Mutations in close to 80 genes have been found to contribute to the pathogenesis of CMT; however, a proportion of patients still lack a confirmed genetic diagnosis.^6^ CMT type 1 (CMT1) is pathologically defined as a demyelinating neuropathy, whereas CMT type 2 (CMT2) is considered axonal. Among these subtypes, CMT1 most frequently results from duplications or deletions involving the peripheral myelin protein 22 (PMP22) gene; these account for an estimated 50%-70% of CMT1 cases.^6^^,^^7^ Other involved genes include myelin protein zero (MPZ), early growth response 2 (EGR2), lipopolysaccharide-induced tumor necrosis factor-alpha factor (LITAF), and neurofilament light (NEFL).^6^^,^^8^^,^^9^

CMT1 typically presents in childhood, causing distal muscle weakness, areflexia, sensory loss, and progressive deformities of the ankles and wrists.^8^^,^^10^^,^^11^ These symptoms gradually worsen, resulting in substantial disability and diminished quality of life (QoL).^7^ Although current management remains supportive—including physical therapy, use of orthopedic devices, and surgical interventions for severe deformities—currently, there are no therapies capable of modifying disease progression. Recent investigational approaches have included gene therapy, strategies targeting axonal transport, restoration of mitochondrial function, and immune- or integrin-based interventions; however, none of these has been proven to effectively prevent or reverse disease progression.^8^^,^^10^^,^^12^ Thus, innovative and transformative CMT treatments are sorely needed, and Schwann cells (SCs), which play a fundamental role in providing trophic support and structural guidance during axonal regeneration, have become an appealing target for therapeutic intervention.^13^ CLZ-2002 is a cell therapy product composed of Schwann-like cells differentiated from tonsil-derived mesenchymal stem cells (T-MSCs). Importantly, CLZ-2002 represents a differentiated cell population that is phenotypically and functionally distinct from the parental T-MSCs. During differentiation, CLZ-2002 exhibits increased expression of CD121a, CD106, and CD112, along with decreased expression of CD26 and CD141. In addition, CLZ-2002 shows enhanced secretion of hepatocyte growth factor (HGF), a key trophic factor involved in Schwann cell-mediated nerve repair. Functional assays demonstrated that CLZ-2002 exhibits greater remyelination and neurite outgrowth-promoting activity than T-MSCs, as well as enhanced immunomodulatory capacity in mixed-lymphocyte reaction assays. Furthermore, CLZ-2002 expresses high levels of Schwann cell-associated genes, including CAD19, GFAP, MBP, NGFR, S100B, and KROX20, suggesting that these cells recapitulate the characteristics of SCs.^14^

Preclinical studies have highlighted the regenerative potential of CLZ-2002. *In vitro* experiments showed that CLZ-2002 facilitated axonal myelination in co-culture with dorsal root ganglion neurons.^14^ Furthermore, in a murine model of sciatic nerve injury, CLZ-2002 significantly improved gait function and promoted axonal regeneration.^2^ Collectively, these findings support the theory that CLZ-2002 has therapeutic potential for treating demyelinating neuropathies such as CMT1.

The biodistribution of CLZ-2002 was also evaluated in BALB/c nu/nu mice following intramuscular administration. Human cells were detected by real-time PCR targeting the human Alu gene in multiple tissues up to twelve weeks after dosing. CLZ-2002 was predominantly detected at the injection site (gastrocnemius muscle), with no meaningful distribution to other organs. These findings suggest that intramuscularly administered CLZ-2002 largely remains localized at the injection site and may exert therapeutic effects within the local tissue microenvironment. Based on this scientific rationale and the encouraging preclinical findings, we conducted a first-in-human Phase 1 clinical trial to evaluate the safety, tolerability, and preliminary efficacy of CLZ-2002 in patients with CMT1.

## Materials and methods

### Study design

This first-in-human, Phase 1, open-label, prospective, dose-escalation study aimed to assess the safety and tolerability of intramuscular CLZ-2002 in patients diagnosed with CMT 1. Regulatory approval was obtained from the Korean Ministry of Food and Drug Safety (Approval No. 101282); ethical approval was granted by the Institutional Review Board of Samsung Medical Center (IRB No. SMC 202-03-065); and the trial was registered at ClinicalTrials.gov (NCT05947578). Recruitment took place at Samsung Medical Center between July 2023 and January 2024, and the study concluded in July 2024.

Participants were assigned to three escalating dose groups: G1 (6 million cells), G2 (12 million cells), and G3 (24 million cells). Each participant received a single dose of CLZ-2002, which was administered via 12 intramuscular injections into both lower extremities. Dose-limiting toxicity (DLT) was defined in accordance with the NCI Common Terminology Criteria for Adverse Events (CTCAEs v5.0). The maximum tolerated dose (MTD) was defined as the highest dose at which <33% of participants experienced a DLT (≤1 of 6 or 2 of 9 participants).

### Patient selection

The study included nine patients aged ≥18 years with both clinical and genetic confirmation of CMT1. Inclusion criteria required weakness of at least foot dorsiflexion and a Charcot–Marie–Tooth Neuropathy Score (CMTNS) ranging from 2 to 30 at screening. Genetic confirmation was established by mutations associated with CMT1A (PMP22), CMT1B (MPZ), CMT1C (LITAF), CMT1D (EGR2), or CMT1F (NEFL).

Among the main exclusion criteria were the presence of other neuromuscular diseases; recent upper or lower limb surgery (within 6 months); serious active infection, such as cellulitis at injection sites; uncontrolled hypertension or diabetes; positive HIV, HBV, or HCV serology; recent history of transient ischemic attack or stroke (within 6 months); diagnosis of malignancy within the past 5 years; or clinically significant respiratory, cardiovascular, renal, gastrointestinal, hepatic, endocrine, hematologic, or psychiatric comorbidities. Women with childbearing potential were excluded if they were pregnant, breastfeeding, or not using adequate contraception.

### Intervention

Tonsillar mesenchymal stem cells (T-MSCs) were isolated and expanded from tonsil tissue harvested from pediatric donors undergoing tonsillectomy. Written informed consent for the tissue donation was obtained according to the research protocol approved by the institutional Review Board (IRB) of Ewha Womans University Mokdong Hospital in Seoul, Korea (IRB No. 2019008-004).

CLZ-2002 consists of Schwann cell-like cells derived from T-MSC. The manufacturing process was performed according to previously established protocols.^2^^,^^14^ See Figure S1 for a schematic overview of the manufacturing workflow and the estimated number of clinical doses that can be generated from a single donor. The final product was provided in AT-vials, each containing 5 million cells suspended in 1.0 mL of CryoStor CS10. The vials were stored in liquid nitrogen tanks at temperatures below –135 °C and transported to the clinical site in validated containers to ensure that this temperature was maintained.

On the day of administration, CLZ-2002 was thawed with a Purple Vie Thawer (Amolifescience Co., Ltd., Republic of Korea) and delivered intramuscularly to the lower extremities using 1 cc syringes and 26-gauge needles. After thawing, the product was kept at room temperature for a maximum of 2 hours and administered within this window. Antihistaminic premedication was administered within 1 hour (±15 minutes) prior to dosing. A topical anesthetic was used at the injection sites, followed by application of an antiseptic for disinfection. Each participant received twelve total intramuscular injections—six in the right lower extremity and six in the left—using sterile, disposable syringes and needles.

### Safety assessment

Safety was designated as the primary endpoint, with all adverse events (AEs) closely monitored via laboratory analyses, physical exams, vital sign measurements, and electrocardiograms throughout the clinical trial (at baseline and at Weeks 1, 4, 12, and 24). All AEs observed were systematically documented for all participants, including the causal relationship with the investigational product and the severity of the reaction.

### Efficacy assessment

The exploratory efficacy of a single intramuscular dose of CLZ-2002 in CMT1 patients was assessed using clinical, imaging, and electrophysiological endpoints (Table S1). Clinical evaluations included the Charcot–Marie–Tooth Neuropathy Score version 2 (Charcot–Marie–Tooth Neuropathy Score—CMTNS; Charcot–Marie–Tooth Exam Score—CMTES; Rasch-modified Charcot–Marie–Tooth Neuropathy Score—CMTNS-R; Rasch-modified Charcot–Marie–Tooth Exam Score—CMTES-R), Overall Neuropathy Limitation Scale (ONLS-leg), Functional Disability Scale (FDS), and 10-Meter Walk Test (10MWT), carried out at baseline and at Weeks 4, 12, and 24. CMTNSv2, evaluated by a neurology specialist, included an examination and assessments of participants’ symptoms and neurophysiology (range: 0-36). The ONLS-leg was used to quantify lower limb disability, and a 1-point difference was considered to reflect a clinically meaningful change.^15^ FDS measured motor deficits, and the 10MWT was used to determine gait speed across a 10-meter distance on a 12-meter walkway.

### MRI

Magnetic resonance imaging (MRI) assessments were performed at both baseline and Week 24, utilizing fat fraction quantification by Ingenia 3.0 T CX (Phillips, New Zealand) in addition to semiquantitative assessment of T1-weighted images according to the Goutallier 5-point scale, where Grade 0 indicated an absence of fat (no fat infiltration), Grade 1 represented slight fat infiltration (with some fatty streaks present), and Grade 2 suggested that there was more muscle than fat (fat muscle).^16–18^

### Electrophysiological assessments

Electrophysiological evaluation consisted of Compound Muscle Action Potential (CMAP), Sensory Nerve Action Potential (SNAP), Motor Nerve Conduction Velocity (MNCV), and Sensory Nerve Conduction Velocity (SNCV) measurements from key motor and sensory nerves using an EMG Relaxograph (Nihon Kohden, Japan) and EMG system 4CH (Naus, WI, USA). Data were collected via surface stimulation and electrodes at baseline and at Weeks 4, 12, and 24.

### Biomarker analysis

Serum samples were collected from patients with CMT type 1 who were enrolled in the CLZ-CMT-101 clinical study. The samples were obtained at baseline and at Weeks 4 and 24 and stored at −80 °C prior to analysis. All biomarker analyses were performed by SML Meditree Co., Ltd. (Seoul, Republic of Korea). Serum concentrations of p75 (NGFR), NCAM-1, and GDF-15 were quantified using commercial sandwich ELISA kits (Human TNFRSF16/NGFR ELISA Kit, KOA0600, Rockland Immunochemicals, Inc.; Human CD56/NCAM-1 ELISA Kit, NCM31-K01, EAGLE Biosciences; Human GDF-15 ELISA Kit, R&D Systems, USA). Absorbance was measured at 450 nm using a microplate reader (BioTek Epoch, Agilent Technologies). Concentrations were calculated using logistic regression based on calculation curves provided by the manufacturers. Samples were analyzed in duplicate or triplicate, with reanalysis performed after appropriate dilution was implemented for values exceeding the upper limit of quantification, in compliance with Good Clinical Laboratory Practice guidelines.

### Statistical analysis

The Safety Set, Full Analysis Set (FAS), and Per Protocol Set (PPS) were utilized for data analysis. FAS was used as the principal population for evaluating efficacy, with PPS providing supportive analysis. Safety evaluations were performed based on the Safety Set. Statistical analyses employed SAS (version 9.4 or higher) and Microsoft Excel. For efficacy evaluation, the last observation carried forward (LOCF) approach was used for participants completing the treatment, and two-sided paired *t*-tests with a 5% significance threshold were used for statistical inference.

Baseline demographic and clinical characteristics were summarized descriptively by group. Safety and tolerability were evaluated by recording TEAEs, AEs, SAEs, ADRs, DLTs, and the corresponding safety assessments (including vital signs, ECGs, and laboratory data). The proportion of patients who experienced TEAEs was determined through Week 24. Efficacy parameters—including CMTNS-v2 (CMTNS, CMTES, CMTNS-R, CMTES-R), ONLS-leg, FDS, and 10MWT—were analyzed descriptively at baseline and at Weeks 4, 12, and 24. Descriptive summaries were also provided for MRI data of proximal lower extremities at baseline and Week 24 (Table S1).

## Results

### Study participants

**Figure 1.**
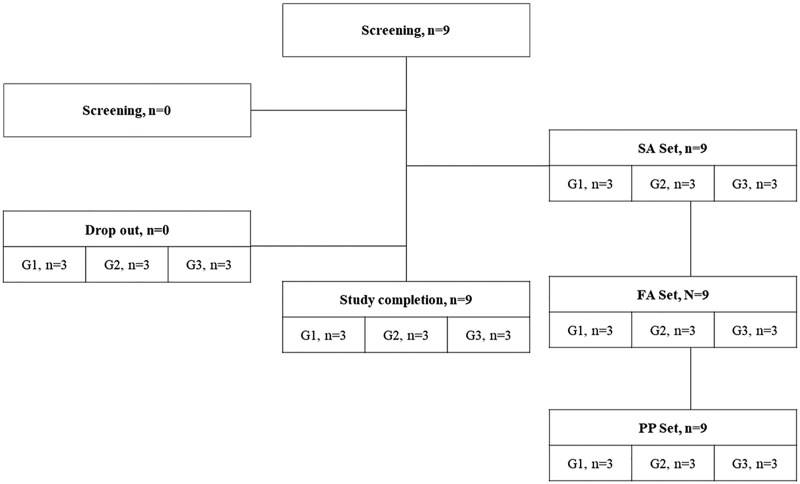
Subject disposition and analysis sets. CMA1 patients were sequentially allocated to each group. SA set—safety analysis set; FA set—full analysis set; PP set—per protocol set; n—subject number.

The subject disposition and analysis sets are listed in Figure 1. Nine participants were screened, all of whom met the eligibility criteria and completed the trial.

### Demographic and other baseline characteristics

In the Safety Set (*n* = 9), 55.56% of participants were female and 44.44% were male. The mean age was 36.67 ± 17.07 years, and there were no statistically significant age differences between groups. The genotype distribution consisted of 55.56% CMT1A (PMP22) and 44.44% CMT1B (MPZ). Within the groups, CMT1B was predominant in G1, whereas CMT1A was more common in G2 and G3. At baseline, 77.78% of patients had moderate disease severity, while 22.22% had severe disease. In G1 and G2, moderate severity was observed in 66.67% (2/3 patients), whereas severe disease was observed in 33.33% (1/3 patients); in G3, all patients (100%, 3/3) had moderately severe disease. The mean disease duration was 117.93 ± 12.54 months, with only minimal variation among groups. The baseline functional assessments were as follows: CMTNS (17.78 ± 3.73), CMTES (13.11 ± 2.80), CMTNS-R (21.22 ± 5.12), CMTES-R (16.11 ± 3.79), ONLS-leg (2.00 ± 0.00), FDS (2.22 ± 0.67), and 10MWT (9.08 ± 1.52 sec). No significant group-wise differences were identified (Table 1).

**Table 1.** Demographic survey and basic characteristics (Safety analysis set, *n* = 9).

	G1 (low dose) (6M cells, *n* = 3)	G2 (medium dose) (12M cells, *n* = 3)	G3 (high dose) (24 M cells, *n* = 3)	All (*n* = 9)
**Gender**				
** Male** (%)	1 (33.33)	1 (33.33)	2 (66.67)	4 (44.44)
** Female** (%)	2 (66.67)	2 (66.67)	1 (33.33)	5 (55.56)
**Age (years)**	37.00 ± 16.64	37.00 ± 24.58	36.00 ± 16.82	36.67 ± 17.07
**CMT type**				
** CMT1A** (%)	1 (33.33)	2 (66.67)	2 (66.67)	5 (55.56)
** CMT1B** (%)	2 (66.67)	1 (33.33)	1 (33.33)	4 (44.44)
**Severity** ^a^				
** Mild** (%)	0 (0.00)	0 (0.00)	0 (0.00)	0 (0.00)
** Moderate** (%)	2 (66.67)	2 (66.67)	3 (100)	7 (77.78)
** Severe** (%)	1 (33.33)	1 (33.33)	0 (0.00)	2 (22.22)
**Disease duration (months)**	120.95 ± 11.18	117.03 ± 19.11	115.81 ± 10.85	117.93 ± 12.54
**CMTNS**	19.67 ± 2.89	17.00 ± 6.08	16.67 ± 1.53	17.78 ± 3.73
**CMTES**	14.67 ± 1.15	12.67 ± 4.62	12.00 ± 1.73	13.11 ± 2.80
**CMTNS-R**	23.00 ± 5.20	20.67 ± 8.14	20.00 ± 2.00	21.22 ± 5.12
**CMTES-R**	17.67 ± 2.89	16.00 ± 6.08	14.67 ± 2.31	16.11 ± 3.79
**ONLS-leg**	2.00 ± 0.00	2.00 ± 0.00	2.00 ± 0.00	2.00 ± 0.00
**FDS**	2.67 ± 0.58	2.33 ± 0.58	1.67 ± 0.58	2.22 ± 0.67
**10MWT (sec/min)**	9.93 ± 1.83	8.10 ± 1.68	9.20 ± 0.72	9.08 ± 1.52

Abbreviations: Charcot–Marie–Tooth neuropathy score (CMTNS); Charcot–Marie–Tooth examination score (CMTES); Rasch–Charcot–Marie–Tooth neuropathy score (CMTNS-R); Rasch–Charcot–Marie–Tooth examination score (CMTES-R); functional disability score (FDS); 10-meter walking test (10MWT); overall neuropathy limitation score-leg (ONLS-Leg).

a The Severity of CMT disease is classified into Mild (less than 10 points), Moderate (10 to 19 points), and Severe (20 points or more) by the CMTNS score.

### Safety evaluations

There were no ADRs, SAEs, SADRs, or DLTs in the Safety Set cohort (Table 2). The overall incidence of TEAEs was 44.44% (4/9 subjects; five cases), with incidence rates of 66.67% (2/3 subjects; three cases) in G2 and 66.67% (2/3 subjects; two cases) in G3; no TEAEs occurred in G1 (Table 2).

**Table 2.** Summary of adverse events.

	G1 (low dose) (6M cells, *n* = 3)	G2 (medium dose) (12M cells, *n* = 3)	G3 (high dose) (24 M cells, *n* = 3)	All (*n* = 9)
**Treatment-emergent adverse event**				
***n* (%)**	0 (0.00)	2 (66.67)	2 (66.67)	4 (44.44)
**95% confidence interval (lower, upper)**	(0.00, 70.76)	(9.43, 99.16)	(9.43, 99.16)	(13.70, 78.80)
**No. of AEs**	0	3	2	5
**Adverse drug reaction^a^**				
***n* (%)**	0 (0.00)	0 (0.00)	0 (0.00)	0 (0.00)
**95% confidence interval (lower, upper)**	(0.00, 70.76)	(0.00, 70.76)	(0.00, 70.76)	(0.00, 33.63)
**No. of AEs**	0	0	0	0
**Serious AE**				
***n* (%)**	0 (0.00)	0 (0.00)	0 (0.00)	0 (0.00)
**95% confidence interval (lower, upper)**	(0.00, 70.76)	(0.00, 70.76)	(0.00, 70.76)	(0.00, 33.63)
**No. of AEs**	0	0	0	0
**Serious adverse drug reaction^a^**				
***n* (%)**	0 (0.00)	0 (0.00)	0 (0.00)	0 (0.00)
**95% confidence interval (lower, upper)**	(0.00, 70.76)	(0.00, 70.76)	(0.00, 70.76)	(0.00, 33.63)
**No. of AEs**	0	0	0	0
**Dose limiting toxicity^b^**				
***n* (%)**	0 (0.00)	0 (0.00)	0 (0.00)	0 (0.00)
**95% confidence interval (lower, upper)**	(0.00, 70.76)	(0.00, 70.76)	(0.00, 70.76)	(0.00, 33.63)
**No. of AEs**	0	0	0	0

It includes a summary of TEAE.

Coding dictionary: MedDRA V27.0.

%: *n*/(No. of subjects of each group in Safety Set) x 100.

a Adverse drug reaction: Adverse events, including their causality of the investigational product, are “Definitely related,” “Probably related,” “Possibly related,” and “Unknown.”

b Dose limiting toxicity: Grade 3 or higher adverse events related to the investigational product based on NCI-CTCAE v5.0. Grade 2 or higher adverse reactions due to infection related to transplanted cells based on NCI-CTCAE v5.0.

The distribution of TEAEs in the Safety Set was as follows: “General disorder and administration site conditions” [22.22% (2/9 subjects, two cases)]; “Musculoskeletal and connective tissue disorders” [11.11% (1/9 subjects; two cases)]; and “Reproductive system and breast disorders” [11.11% (1/9 subjects; one case)] (Table 3). No treatment-related serious adverse events were recorded, and all TEAEs were of mild (Grade 1) or moderate (Grade 2) severity (Table 3).

**Table 3.** Treatment-emergent adverse event by SOC/PT.

System organ class preferred term	Treatment-emergent adverse events, *n* (%), [No. of AEs]			
	G1 (low dose) (6M cells, *n* = 3)	G2 (medium dose) (12M cells, *n* = 3)	G3 (high dose) (24 M cells, *n* = 3)	All (*n* = 9)
**General disorders and administration site conditions**	0 (0.00), [0]	1 (33.33), [1]	1 (33.33), [1]	2 (22.22), [2]
** Injection site pain**	0 (0.00), [0]	1 (33.33), [1]	1 (33.33), [1]	2 (22.22), [2]
**Musculoskeletal and connective tissue disorders**	0 (0.00), [0]	1 (33.33), [2]	0 (0.00), [0]	1 (11.11), [2]
** Osteoarthritis**	0 (0.00), [0]	1 (33.33), [1]	0 (0.00), [0]	1 (11.11), [1]
** Rotator cuff syndrome**	0 (0.00), [0]	1 (33.33), [1]	0 (0.00), [0]	1 (11.11), [1]
**Reproductive system and breast disorders**	0 (0.00), [0]	0 (0.00), [0]	1 (33.33), [1]	1 (11.11), [1]
*** Vulvovaginal pruritus***	0 (0.00), [0]	0 (0.00), [0]	1 (33.33), [1]	1 (11.11), [1]
**Total**	0 (0.00), [0]	2 (66.67), [3]	2 (66.67), [2]	4 (44.44), [5]

It includes a summary of TEAE.

Coding dictionary: MedDRA V27.0.

%: *n*/(No. of subjects of each group in Safety Set) × 100.

### Charcot–Marie–Tooth neuropathy score

Based on the CMTNSv2 data, CMTNS, CMTES, CMTNS-R, and CMTES-R were assessed for changes from baseline. The CMTNS decreased by 7.00 ± 1.00 (*P* < .01) in the low-dose group (G1, 6 million cells), 5.33 ± 1.53 (*P* < .05) in the medium-dose group (G2, 12 million cells), and 4.00 ± 1.00 (*P* < .05) in the high-dose group (G3, 24 million cells) compared with the baseline. In all subjects, CMTNS was reduced by 5.44 ± 1.67 (*P* < .001) (Table 4). For CMTES, the following reductions from the baseline were reported: 7.33 ± 0.58 (*P* < .05) in G1, 5.00 ± 1.00 (*P* < .05) in G2, and 3.67 ± 0.58 (*P* < .01) in G3. The overall decrease in CMTES for all subjects was 5.33 ± 1.73 (*P* < .001). CMTNS-R showed a decrease from baseline to 6.33 ± 1.53 (*P* < .05) in G1, 5.00 ± 1.00 (*P* < .05) in G2, and 4.33 ± 0.58 (*P* < .05) in G3. In the entire cohort, CMTNS-R decreased from baseline to 5.22 ± 1.30 (*P* < .001) (Table 4). For CMTES-R, we noted the following reductions from the baseline: 6.67 ± 1.53 (*P* < .05) in G1, 5.00 ± 1.00 (*P* < .05) in G2, and 6.67 ± 1.53 in G3. CMTES-R was reduced by 5.11 ± 1.76 (*P* < .001) in all subjects (Table 4).

**Table 4.** Changes of CMTES, CMTNS, CMTES-R, and CMTNS-R scores.

	G1 (low dose) (6M cells, *n* = 3)	G2 (medium dose) (12M cells, *n* = 3)	G3 (high dose) (24 M cells, *n* = 3)	All (*n* = 9)
**CMTNS**				
**Baseline**	19.67 ± 2.89	17.00 ± 6.08	16.67 ± 1.53	17.78 ± 3.73
**Week 24**	12.67 ± 3.79	11.67 ± 6.66	12.67 ± 2.31	12.33 ± 4.03
**Difference (Week 24-Baseline)**	−7.00 ± 1.00	−5.33 ± 1.53	−4.00 ± 1.00	−5.44 ± 1.67
***P*-value (paired t-test)**	.0068^**^	.0263*	.0203*	.0001^***^
**CMTES**				
**Baseline**	14.67 ± 1.15	12.67 ± 4.62	12.00 ± 1.73	13.11 ± 2.80
**Week 24**	7.33 ± 1.53	7.67 ± 4.73	8.33 ± 2.08	7.78 ± 2.73
**Difference (Week 24-Baseline)**	−7.33 ± 0.58	−5.00 ± 1.00	−3.67 ± 0.58	−5.33 ± 1.73
***P*-value (paired t-test)**	.0021^**^	.0131*	.0082^**^	.0001^****^
**CMTNS-R**				
**Baseline**	23.00 ± 5.20	20.67 ± 8.14	20.00 ± 2.00	21.22 ± 5.12
**Week 24**	16.67 ± 5.69	15.67 ± 7.37	15.67 ± 1.53	16.00 ± 4.74
**Difference (Week 24-Baseline)**	−6.33 ± 1.53	−5.00 ± 1.00	−4.33 ± 0.58	−5.22 ± 1.30
***P*-value (paired t-test)**	.0189*	.0131*	.0059^**^	.0001^****^
**CMTES-R**				
**Baseline**	17.67 ± 2.89	16.00 ± 6.08	14.67 ± 2.31	16.11 ± 3.79
**Week 24**	11.00 ± 3.00	11.00 ± 5.29	11.00 ± 1.00	11.00 ± 3.08
**Difference (Week 24-Baseline)**	−6.67 ± 1.53	−5.00 ± 1.00	−3.67 ± 1.53	−5.11 ± 1.76
***P*-value (paired t-test)**	.0171*	.0131*	.0533	.0001^****^

* *P* < .05;

** *P* < .01;

*** *P* < .001;

**** *P* < .0001.

**Figure 2.**
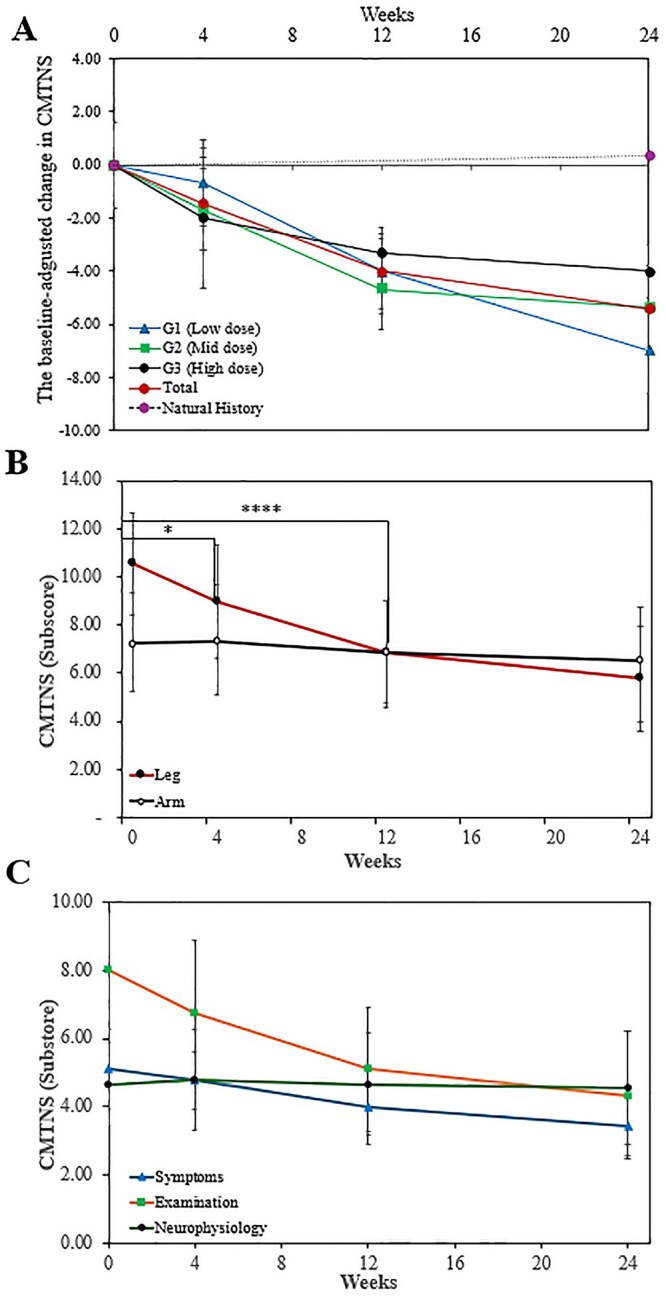
Longitudinal assessment of Charcot–Marie–Tooth neuropathy score v2 (CMTNSv2) over Week 24. (A) Adjusted mean changes in CMTNSv2 over Week 24 for each group are compared with the expected mean change over Week 24 derived from published natural history data.^19^ (B) Mean CMTNSv2 scores for the upper and lower limbs were analyzed by disaggregating upper and lower limb scores recorded over Week 24. The differences in CMTNSv2 scores for the lower limbs across all follow-up visits compared to baseline were statistically significant (*P < .05). (C) Mean CMTNSv2 domain scores (symptoms, examination, and neurophysiology) at baseline and follow-up visits over Week 24. Error bars denote *SD*.

The statistical comparisons for CMTNS, CMTES, CMTNS-R, and CMTES-R at baseline and at Week 24 are summarized in Table 4. All four scores showed statistically significant decreases from baseline in the overall cohort, with mean reductions of 5.44 ± 1.67 (*P* < .001), 5.33 ± 1.73 (*P* < .001), 5.22 ± 1.30 (*P* < .001), and 5.11 ± 1.76 (*P* < .001), respectively. Furthermore, the CMTNSs measured at Week 4, 12, and 24 demonstrated a consistent downward trend from baseline across all groups. The reduction in CMTNS observed in patients receiving CLZ-2002 is notable in light of previous reports that reported an average annual increase of 0.687 points in CMTNS among patients with CMT1 (Figure 2A).^19^

When CMTNS was evaluated separately for upper and lower limbs, the lower limb scores showed a statistically significant reduction from baseline (10.56 ± 2.13) at Week 4 (9.00 ± 2.35, *P* < .05), Week 12 (6.89 ± 2.15, *P* < .001), and Week 24 (5.78 ± 2.17, *P* < .05) after administration of CLZ-2002. In contrast, upper limb scores showed a downward trend from the baseline (7.22 ± 1.99) to Week 4 (7.33 ± 2.24), Week 12 (6.89 ± 2.32), and Week 24 (6.56 ± 2.60), but the changes were not statistically significant (Figure 2B).

When the CMTNS domains were analyzed separately, improvements were primarily observed in the symptoms and examination domains. The symptom domain decreased progressively from 5.11 ± 1.17 at baseline to 3.44 ± 0.88 at Week 24, while the examination domain declined from 8.00 ± 1.73 to 4.33 ± 1.87 over the same period. In contrast, the neurophysiology domain remained relatively stable throughout the study period (4.67 ± 1.22 at baseline vs. 4.56 ± 1.67 at Week 24), showing only minor fluctuations over time. Detailed data showcasing the domain-specific changes in CMTNS are provided in Figure 2C and Table S3. These findings suggest that the overall improvement in CMTNS was mainly driven by improvements in patient-reported symptoms and physician-assessed neurological examination findings.

### FDS and ONLS-leg score

**Figure 3.**
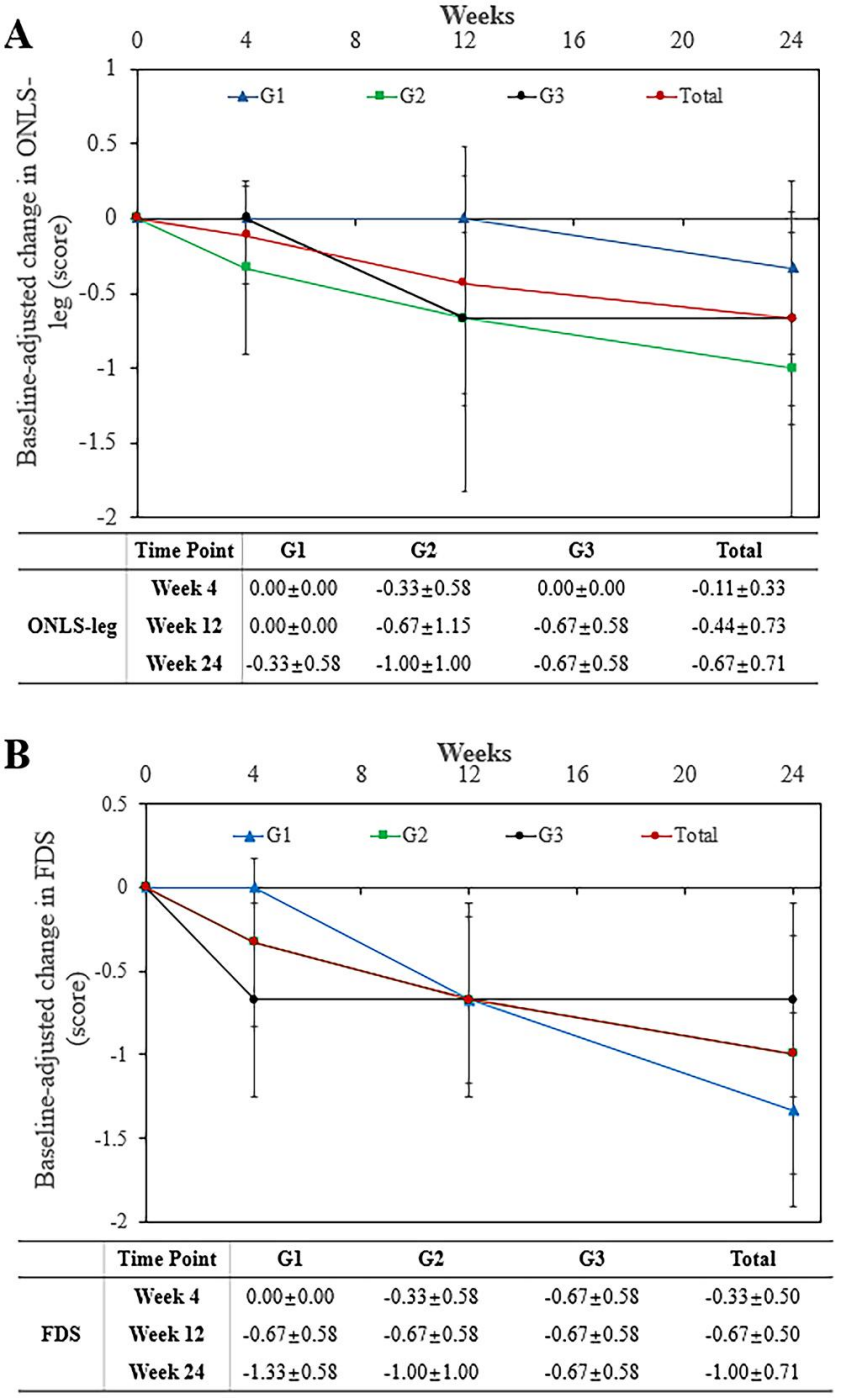
Trends in the overall neuropathy limitations scale (ONLS-leg) and functional disability scale (FDS) across Week 24. (A) ONLS-leg scores were measured at baseline and at Weeks 4, 12, and 24. (B) FDSs were evaluated at baseline and in Weeks 4, 12, and 24. Error bars correspond to *SD*.

We assessed ONLS-leg and FDSs to characterize the longitudinal functional changes in subjects receiving CLZ-2002. Compared to baseline, the ONLS-leg score in the nine subjects receiving CLZ-2002 decreased by 0.11 ± 0.33, 0.44 ± 0.73, and 0.67 ± 0.71 points at Weeks 4, 12, and 24, respectively. Similarly, the FDS decreased by 0.33 ± 0.50, 0.67 ± 0.50, and 1.00 ± 0.71 points at Weeks 4, 12, and 24 relative to baseline (Figure 3). At Week 24 post-administration of CLZ-2002, the mean changes in ONLS-leg score from baseline were −0.33 ± 0.58 in G1, −1.00 ± 1.00 in G2, and −0.67 ± 0.58 in G3. The corresponding mean changes in FDS were −1.33 ± 0.58 points in G1, −1.00 ± 1.00 points in G2, and −0.67 ± 0.58 points in G3. This indicates that ONLS-leg and FDS declined from baseline in every group (Figure 3). As an exploratory analysis, we also performed the 10MWT (10-meter walking test), in which we measured both the average time and speed of a 10 m walk completed by all subjects to evaluate differences between the baseline and Week 24. No clinically significant changes were identified in any group.

### MRI and electrophysiology

Exploratory evaluations, including MRI and electrophysiological assessments, showed no significant changes from baseline to Week 24. Most participants exhibited limb muscle MRI grades between 0 and 2, which remained stable throughout the treatment period. Similarly, no detectable alterations were observed in the electrophysiological parameters, including CMAP, SNAP, MNCV and SNCV, across the evaluated nerves.

### Biomarkers

p75, NCAM1, and GDF15—all of which have been reported to be elevated in the blood of CMT patients^20^—were analyzed as exploratory circulating biomarkers to support the efficacy assessment. The plasma concentrations of these biomarkers were measured in participants with demyelinating CMT blood samples collected before and after CLZ-2002 administration.

**Figure 4.**
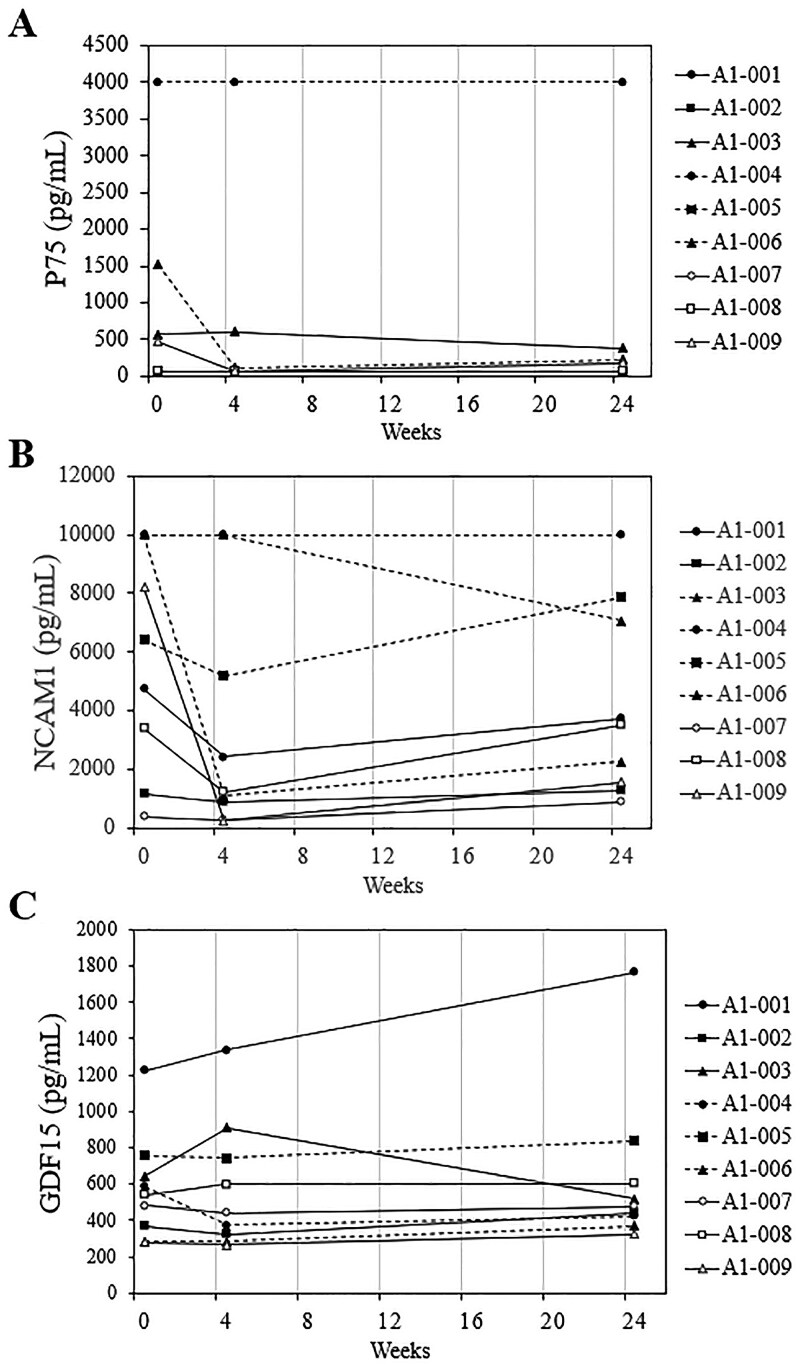
Sequential changes in plasma concentrations of p75, NCAM1, and GDF15 at baseline and at Weeks 4 and 24. (A) p75 plasma concentrations at baseline and at Weeks 4 and 24; (B) NCAM1 plasma concentrations at baseline and Weeks 4 and 24; (C) GDF15 plasma concentrations at baseline and at Weeks 4 and 24.

The plasma concentration of p75 showed substantial inter-individual variability, and the overall trend could not be clearly established because several measurements either fell below or exceeded the assay’s detection limit. For subject A1-006, the p75 concentration was 1,523.58 pg/mL prior to CLZ-2002 administration. It decreased to 120.69 pg/mL after Week 4 of treatment and then increased modestly to 223.12 pg/mL after Week 24 compared to the Week 4 measurement. For subject A1-009, the p75 plasma level was 475.36 pg/mL before treatment but dropped below the detection limit after Week 4 of CLZ-2002 administration, subsequently showing a slight increase to 183.54 pg/mL after Week 24 (Figure 4A). The blood concentration of NCAM1 decreased after Week 4 of CLZ-2002 administration in all subjects except A1-003 and A1-004, in whom analysis was limited by values exceeding the detection limit. At Week 24, NCAM1 levels relative to the Week 4 (Figure 4B). The plasma concentration of GDF15 did not demonstrate any marked changes at baseline, Week 4, or Week 24 after CLZ-2002 administration (Figure 4C).

These findings align with prior reports highlighting NCAM1 as a potential biomarker for CMT1 disease.^20^ The CMTNS (17.78 ± 3.73 points) and baseline blood concentrations of NCAM1 were 351.94 ∼ 8197.07 pg/mL, excluding values above the upper limit of quantification (ULOQ). At Week 4 after CLZ-2002 administration, the CMTNSv2 score decreased by 0-5 points; this was accompanied by reductions in NCAM1 blood concentration across participants. By Week 24, the CMTNS maintained a 3-8 point reduction compared with the baseline, while NCAM1 concentrations showed a partial increase toward baseline levels.

**Figure 5.**
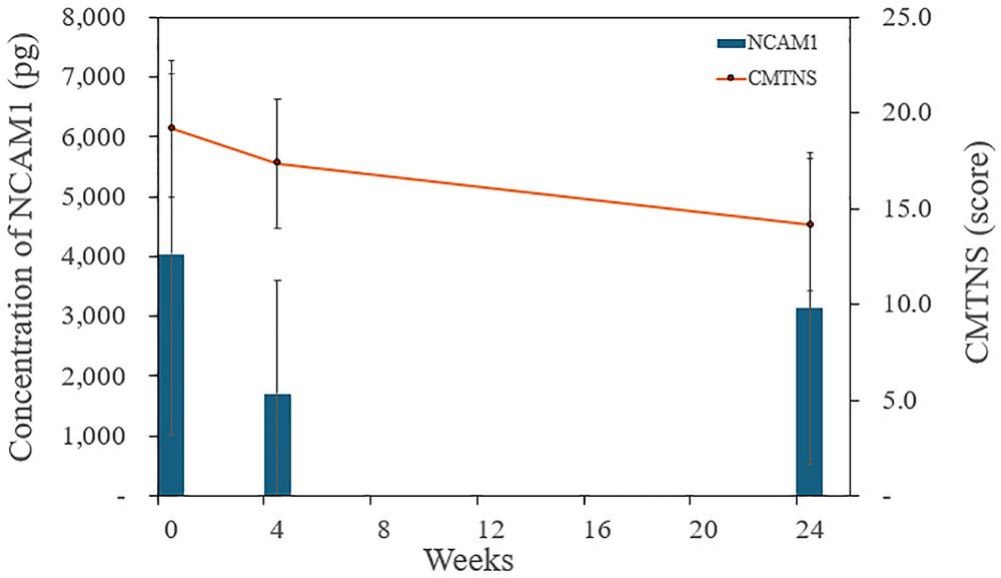
Correlation between CMTNSv2 score and NCAM1 concentration in CMT patients. NCAM1 concentrations were compared with CMTNSv2 scores to evaluate the relationship between the biomarker level and disease severity. Each data point presents an individual subject, and error bars indicate *SD*. Subjects A1-003, A1-004, and A1-006 were excluded from the analysis because their NCAM1 concentrations exceeded the upper limit of quantification (ULOQ; 10 000 pg/mL). The individual subject data underlying this analysis are provided in Figure S5.

To further explore the relationship between disease severity and circulating biomarkers, the association between total CMTNSs and NCAM1 plasma concentrations was evaluated. Subjects A1-003, A1-004, and A1-006, whose NCAM1 concentrations exceeded the upper limit of quantification (ULOQ, 10 000 pg/mL), were excluded from the correlation analysis. Using the remaining data set, CMTNSv2 scores were plotted against NCAM1 plasma concentrations (Figure 5); the corresponding raw data are provided in Table S4. These observations suggest a temporal association between changes in NCAM1 concentrations and the clinical outcome measured by CMTNSv2.

## Discussion

Though there have been significant developments in our understanding of the molecular pathogenesis of CMT, thus far, no disease-modifying therapies have been established. A range of potential strategies has been explored, including pharmacological agents, gene-based approaches, and regenerative therapies. One possible pharmacological approach involves the combination drug PXT3003, which has been evaluated in several clinical trials for CMT1A; however, it has not consistently demonstrated clinical benefit in confirmatory studies.^8^ Gene-based strategies, including plasmid-based gene therapies such as VM202, have also been explored.^8^ In addition, early clinical investigations of regenerative or cell-based therapies, such as EN001, have been initiated. However, clinical evidence in support of these strategies remains limited, and thus far no therapy has demonstrated robust disease-modifying efficacy in CMT.

In this context, Schwann cell-based therapy represents a biologically rational strategy for CMT, as these cells play a central role in peripheral nerve myelination and regeneration. Preclinical studies, including our previous study in C22 mice, demonstrated that Schwann cell-like cells derived from hT-MSCs (CLZ-2002) can promote peripheral nerve regeneration and remyelination, resulting in functional improvement in demyelinating neuropathies.^2^ Consistent with these findings, biodistribution analysis in BALB/c nu/nu mice demonstrated that CLZ-2002 cells were predominantly detected at the injection site (gastrocnemius muscle) for several weeks following intramuscular administration, whereas they were not consistently detected in other tissues (Table S5).

These findings are consistent with previous reports showing that MSC-derived cells delivered intramuscularly tend to persist locally rather than being systematically distributed. Previous studies have demonstrated that MSCs administered via the intramuscular route remain localized at the injection site with minimal systemic distribution.^21–23^ According to Creane et al., intramuscularly injected MSCs were detected exclusively at the injection site and were not observed in major organs, including the lungs, liver, spleen, or brain.^21^ Similarly, Cai et al. demonstrated that labeled MSCs remained confined to the injected muscle without evidence of systemic biodistribution.^22^ A review by Hamidrian Jahromi et al. provides further evidence that intramuscularly administered MSCs predominantly exhibit localized retention with minimal migration into systemic circulation.^23^

This localized persistence suggests that the therapeutic effects of CLZ-2002 may be mediated through the secretion of neurotrophic factors and cytokines that support nerve repair and remyelination within the surrounding tissue microenvironment. Importantly, CLZ-2002 is not a conventional MSC product but a Schwann cell-like cell differentiated from hT-MSCs; therefore, it is mechanistically and functionally distinct from naïve MSCs. While conventional MSCs primarily exert their therapeutic effects through immunomodulatory and paracrine mechanisms,^24–26^ CLZ-2002 recapitulates the key functional properties of Schwann cells, including their direct involvement in myelination and peripheral nerve regeneration.^27^ Accordingly, the therapeutic effects of CLZ-2002 are likely mediated not only by paracrine signaling but also by direct contribution to nerve repair through Schwann cell-like activity. T-MSCs have also been reported to exhibit enhanced proliferative capacity and immunomodulatory properties compared with MSCs from other sources, suggesting their potential utility as a cell source for therapeutic applications.^25^ Collectively, these findings support a mechanism in which CLZ-2002 enhances nerve regeneration through localized Schwann cell activity and trophic support.

Against this mechanistic background, this study is the first-in-human clinical trial to evaluate the safety and preliminary efficacy of CLZ-2002, a Schwann cell-like cell therapy designed to enhance remyelination, neuroprotection, and nerve regeneration in patients with Charcot–Marie–Tooth disease type 1 (CMT1).^13^^,^^28^ Intramuscular administration of CLZ-2002 was well tolerated and did not result in any treatment-emergent adverse events (TEAEs), dose-limiting toxicities (DLTs), or adverse drug reactions (ADRs). All observed adverse events (AE) were mild or moderate in severity, supporting progression to larger clinical trials and establishing 24 million cells per patient as the maximum tolerated dose (MTD).

Although safety was the primary focus of this trial, exploratory analyses of efficacy were encouraging. The CMTNS, a widely recognized measure for tracking disease progression, deteriorated by 0.3 to 0.7 points annually in natural history studies.^19^^,^^29^ In comparison, patients receiving CLZ-2002 experienced significant CMTNS reductions over Week 24, with mean decreases of –7.00 ± 1.00, –5.33 ± 1.53, and –4.00 ± 1.00 points in the low-, medium-, and high-dose groups, respectively, and an overall decline of –5.44 ± 1.67 points. Given that CMT typically exhibits gradual and progressive deterioration, the magnitude of improvement observed within six months suggests a potentially meaningful therapeutic effect.

Notably, the low-dose group exhibited the greatest clinical improvement. This may be attributed to the local microenvironmental effects of CLZ-2002. Biodistribution data indicated that the cells remained predominantly at the injection site, suggesting that therapeutic efficacy is mediated primarily through the secretion of neurotrophic factors and other regenerative proteins released by the transplanted cells, rather than systemic engraftment. At higher doses, excessive cell density may disrupt local cell-to-cell signaling or alter the microenvironment, potentially inducing mild inflammatory responses, which could lead to a plateau or slight reduction in functional benefits.^30^ Furthermore, the greater improvements observed in the injected lower extremities support a regional therapeutic effect, which may be associated with localized inhibition of demyelination and enhanced remyelination.

Other functional assessments indicated similar trends consistent with clinical improvement. The FDS decreased by approximately 1 to 2 points in most patients, reflecting improvements in gait performance and reduced fatigue, while the overall neuropathy limitation scale for the lower extremities (ONLS-leg) revealed small but clinically meaningful reductions, as a 1-point change is considered clinically relevant in CMT clinical trials.^15^ The 10-meter walk test did not show significant changes, likely due to the mild impairment of the study cohort, all of whom were able to walk independently at baseline.

MRI of the thigh muscles did not reveal significant changes in fat infiltration or Goutallier grade over the observation period in Week 24. This finding is consistent with previous studies demonstrating that structural muscle changes in CMT progress slowly, suggesting that longer follow-up periods may be required to detect meaningful alterations. Similarly, electrophysiological assessments failed to show consistent changes, which may reflect baseline variability and these tests’ limited sensitivity for detecting short-term therapeutic effects.

Recently, a significant number of clinical trials have been initiated to develop treatments for CMT. In Pharnext’s Phase 2 clinical trial (NCT01401257), it was demonstrated that administering high-dose PXT3003 to 19 CMT1A patients twice daily for 12 months resulted in a 0.6-point reduction in CMTNS from baseline, alongside a 0.1-point increase in ONLS-leg from baseline.^31^ Furthermore, in a Phase 3 clinical trial (NCT02579759) reported in 2021, the CMTES was reduced by 0.49 points in the placebo group, 0.48 points in the low-dose group, and 0.54 points in the high-dose group after 15 months when compared to the baseline. The ONLS-leg metric increased by 0.14 ± 0.59 points in the placebo group, whereas it decreased by 0.05 ± 0.49 points in the low-dose group and by 0.07 ± 0.43 points in the high-dose group. Despite these findings, the PREMIER trial, mandated by the FDA as a confirmatory study (NCT0476275), failed to achieve its primary endpoints according to a press release from the manufacturer.^8^^,^^32^ Helixmith’s Phase 1/2a clinical trial of VM202 and ENCell’s Phase 1 clinical trial of EN001 have both been completed; however, no outcome data have been disclosed publicly to date.

Compared with efficacy test scores reported in prior clinical trials, our trial demonstrated more substantial effects: CMTNS decreased by 5.44 ± 1.67 points, CMTES by 5.33 ± 1.73 points, and ONLS-leg by 0.67 ± 0.7 points. These outcomes suggest that CLZ-2002 could be developed as a disease-modifying therapy for CMT1.

In conclusion, we carried out the first clinical investigation (first-in-human trial) utilizing Schwann cells in patients diagnosed with CMT1. Our results demonstrate the absence of ADRs, SAEs, SUSARs, and DLT following a single intramuscular injection of CLZ-2002, establishing an MTD of up to 24 million cells/4.8 mL. The data further support the efficacy of CLZ-2002 with regard to key assessment endpoints such as CMTNS, CMTES, CMTNS-R, CMTES-R, ONLS-leg, and FDS in CMT1 patients. An analysis of the relationship between NCAM1 and CMTNSs provided indirect evidence for the remyelination activity and duration of therapeutic effect of CLZ-2002. Nonetheless, the limited sample size restricts the statistical power of these findings. As a result, we intend to pursue a Phase 2 clinical study involving a more extensive patient cohort to validate the therapeutic effects of CLZ-2002.

## Conclusion and summary

This first-in-human clinical trial assessed the safety and exploratory efficacy of CLZ-2002, a Schwann cell-like therapy for patients with CMT1. Intramuscular administration demonstrated acceptable tolerability, with no reported treatment-related SAEs, DLTs, or ADRs, and only mild-to-moderate TEAEs. Preliminary efficacy results indicated that clinically relevant improvements in CMTNS, ONLS-leg, and FDSs occurred during Week 24. The CMTNS showed substantial changes that surpassed those typically observed in natural history studies. Improvements were especially noticeable in the injected lower extremities, indicating a localized therapeutic benefit likely mediated by enhanced remyelination and neuroprotection. While MRI and electrophysiological measures did not reveal uniform changes, plasma NCAM1 reductions support its utility as a potential treatment response biomarker. Despite the small sample size and open-label study design, these results provide preliminary evidence of both the safety and disease-modifying effects of CLZ-2002, emphasizing the need for further evaluation in larger controlled trials.

## Supplementary Material

szag048_Supplementary_Data

## Data Availability

The data underlying this article cannot be made publicly available due to privacy concerns. De-identified data may be provided by the corresponding author upon reasonable request and with appropriate institutional approval.
