# Supplementary material for: A Phase 1 clinical trial to evaluate the safety and tolerability of CLZ-2002 for the treatment of patients with Charcot–Marie–Tooth disease type 1
Source: Stem Cells Transl Med. 2026 Jul 28;15(8):szag048. doi: 10.1093/stcltm/szag048 (PMC13415455; doi:10.1093/stcltm/szag048)
Supplement: szag048_Supplementary_Data [file szag048_supplementary_data.docx]

**SUPPLEMENTARY INFORMATION**

# **Title**

A Phase 1 Clinical Trial to Evaluate the Safety and Tolerability of CLZ-2002 for the Treatment of Patients with Charcot-Marie-Tooth Disease type 1

# **Author**

Hyesun Kim^1,‡^, Byung-Ok Choi ^2,‡,^*, Hyunju Lee^1^, Saeyoung Park^3^, Jaeseung Lim^1^, Sung-Chul Jung^3,^*

^1^ Cellatoz Therapeutics Inc., Seongnam-si, Gyeonggi-do 13487, Republic of Korea

^2^ Department of Neurology, Samsung Medical Center, Sungkyunkwan University School of Medicine, Seoul 06351, Republic of Korea

^3^ Department of Biochemistry, College of Medicine, Ewha Womans University, Seoul 07804, Republic of Korea

* Correspondending authors: Byung-Ok Choi, MD, PhD (bochoi77@hanmail.net) and Sung-Chul Jung, MD, PhD (jungsc@ewha.ac.kr)

‡ These authors contributed equally to this work.

**Supplementary Table 1. Summary of Study Flow Chart**

|  | **SC** | **BL** | | | **Follow-up** | | | **COV** | **UV** |
| --- | --- | --- | --- | --- | --- | --- | --- | --- | --- |
| **Visit** | **V1** | **V2*** | | | **V3** | **V4** | **V5** | **V6** |  |
| **Time of**  **Visit** | **- 4 w ~** | **D1** | | | 1w ±3D | 4w±7D | 12w±7D | 24w±14D | If needed |
|  |  | Pre-dose  -7D~ | Dosing | Post dose |  |  |  |  |  |
| Informed Consent ^1)^ | √ |  |  |  |  |  |  |  |  |
| Inclusion/Exclusion Criteria | √ | √ |  |  |  |  |  |  |  |
| Subject Basic Information^2)^ | √ |  |  |  |  |  |  |  |  |
| Medical history^3)^ | √ | √ |  |  |  |  |  |  |  |
| Prior/concomitant medications^3)^ | √ | √ |  |  | √ | √ | √ | √ | √ |
| Physical examination^4)^ | √ | √ |  |  | √ | √ | √ | √ | √ |
| Vital signs | √ | √ |  | √ | √ | √ | √ | √ | √ |
| Laboratory tests^5)^ | √ | √ |  |  | √ | √ |  | √ | (√) |
| Urinalysis^6)^ | √ | √ |  |  | √ | √ |  | √ | (√) |
| Pregnancy test^7)^ | √ |  |  |  |  |  |  | √ |  |
| Chest X-ray |  | √ |  |  |  |  |  | √ | (√) |
| Electrocardiogram (12-lead ECG) |  | √ |  |  |  |  |  |  | (√) |
| Neurological examination |  | √ |  |  | √ | √ | √ | √ | (√) |
| CMTNS-v2 ^8)^ | √ | √ |  |  |  | √ | √ | √ | (√) |
| ONLS |  | √ |  |  |  | √ | √ | √ | (√) |
| FDS |  | √ |  |  |  | √ | √ | √ | (√) |
| 10MWT |  | √ |  |  |  | √ | √ | √ | (√) |
| MRI leg |  | √ |  |  |  |  |  | √ |  |
| CMAP |  | √ |  |  |  | √ | √ | √ | (√) |
| SNAP |  | √ |  |  |  | √ | √ | √ | (√) |
| MNCV |  | √ |  |  |  | √ | √ | √ | (√) |
| SNCV |  | √ |  |  |  | √ | √ | √ |  |
| Use or not of assistive devices | √ | √ |  |  | √ | √ | √ | √ | √ |
| Investigational product Injection |  |  | √ |  |  |  |  |  |  |
| Check for Adverse Events^9)^ |  | √ | √ | √ | √ | √ | √ | √ | √ |
| Measurement of Biochemical Biomarkers^10)^ |  | √ |  |  |  | √ |  | √ |  |
| HLA Typing |  | √ |  |  |  |  |  |  |  |
| HLA Antibody Test |  | √ |  |  |  | √ |  |  |  |
| Long-term Follow-up^11)^ |  |  |  |  |  |  |  |  |  |

*Subjects were admitted prior to investigational product administration on Visit 2 and were discharged after hospitalization for 2 days and 3 nights but the hospital stay may have changed depending on investigator’s discretion.

1. A written ICF was obtained prior to conducting any clinical trial procedures.
2. After obtaining a written ICF, demographic information (initials, gender, date of birth, age, history of smoking, history of alcohol intake, and race) was investigated.
3. Prior medications/medical history was collected before the injection of the investigational product.

For prior medications, drugs currently being taken and the history of drugs taken within 6 months prior to screening (Visit 1) and immediately until the injection of the investigational product was checked. The medical history was regarded as a disease history within 12 months prior to screening (Visit 1), and medical histories that fell under the exclusion criteria including a cancer history was checked for the relevant period.

However, all information related to CMT1 (including subject’s prior and concomitant medications, age of onset, symptoms at onset, and family history) was collected regardless of the period.

1. A physical examination was performed on subject’s height, weight, head and ears, nose, throat, eyes, gastrointestinal system, hepatobiliary system, cardiovascular system, respiratory system, nervous system, endocrine system, musculoskeletal system, immune system, blood and lymph system, skin, urinary system, and reproductive system.

*Height, weight, and BMI were collected and analyzed as vital signs.

1. If the subject already had results within 4 weeks of the screening visit, the results were used as screening test values. Screening, Baseline, V4, and V6 were kept on an empty stomach for at least 8 hours

- Hematology: WBC, PLT, Hb, Hct, RBC, ANC
- Blood chemistry: sodium, potassium, chloride, carbon dioxide, glucose, BUN, Cr, calcium, phosphorus, magnesium, uric acid, TP, albumin, creatine phosphokinase, TB, direct bilirubin, AST, ALT, gamma-glutamyl transferase, alkaline phosphatase, cholesterol and triglycerides, LDL, HDL, HbA1c, eGFR. (HbA1c and eGFR test were measured only at screening)
- Blood coagulation: aPTT, PT (they were measured only at screening and at the last visit.)
- Serum Immunology: Anti- HIV, HBV (HBsAg), HBV (Anti-HBV), Anti- HCV, VDRL (they were measured only at screening and at the last visit.)

1. If the subject already had results within 4 weeks of the screening visit, the results were used as screening test values.

- Urinalysis: Specific gravity, pH, glucose, protein, nitrites, bilirubin, ketones, leukocytes

1. A urine HCG pregnancy test was performed only in female subjects of childbearing potential. If a urine test was not possible, it can be replaced with a blood test.
2. After performing the CMTNS-v2 test, we analyzed CMTNS and CMTES, and weighted CMTNS-R and CMTES-R.
3. Adverse events were investigated and recorded from the time of informed consent until the last visit and were additionally checked up to 30 minutes immediately after the injection of the investigational product at Visit 2.

* In case of drop-out (early termination), the same tests and evaluations as the close-out visit were performed.

1. Blood for biomarker analysis was collected after keeping an empty stomach for 8 hours
2. Long-term follow-up (separate protocol applied) was expected to be conducted for 5 years (12M, 24M, 36M, 48M and 60M) after administration, and delayed adverse reactions (tumor formation) were confirmed through phone calls or visits. The results of this clinical trial were reported as a clinical study report after Visit 6, and information collection and reporting were carried out separately for the long-term follow-up. The information collected was limited to survival or the occurrence of SAEs (e.g., abnormalities in blood, immunity, nerves, tumors, and others). For additional tests, visits were performed under the investigator’s discretion.

**Supplementary Figure 1. Manufacturing process and production scalability of CLZ-2002.**

**
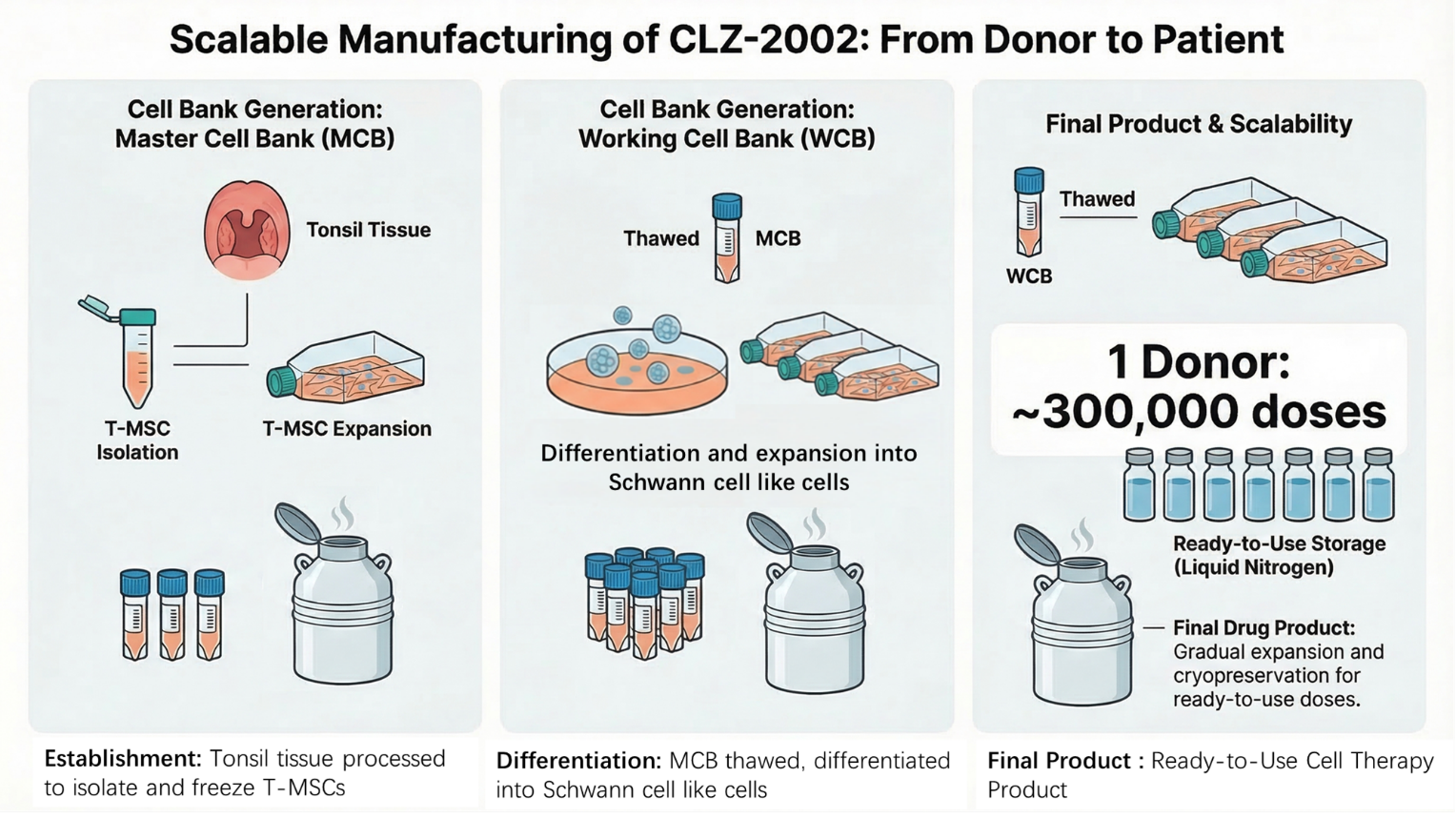
**

**Supplementary Table 2. The specification of the CLZ-2002**

| **Test** | | **Acceptance Criteria** |
| --- | --- | --- |
| Appearance | | A milky white frozen cell suspension is filled in a transparent 2mL polypropylene vial. |
| Sterility | | Negative |
| Mycoplasma | | Negative |
| Adventitious virus | | Negative |
| Endotoxin | | <0.5EU/mL |
| Cell morphology | | Neuron-specific morphology with bipolar or tripolar dendritic projections. |
| Total cell count | | 4~6×10^6^cells/vial |
| Cell viability | | ≥80% |
| Identity | CD121a | ≥80% |
|  | CD9 | ≥80% |
|  | CD140a | ≥80% |
|  | CD166 | ≥80% |
| Purity | CD14 | <5% |
|  | CD34 | <5% |
|  | CD118 | <5% |
|  | BSA | <1ug/mL |
|  | Gentamicin | <10ug/mL |
| Potency | HGF | ≥3,000pg/mL |

**Supplementary Table 3. Individual Patient-Level CMTNSv2 Domain Scores (Symptoms, Examination, and Neurophysiology) at baseline and Follow-up Visits.**

|  | Subject No. | BLV | Week 4 | Week 12 | Week 24 |
| --- | --- | --- | --- | --- | --- |
| **Symptoms** | A1-001 | 7 | 6 | 6 | 4 |
|  | A1-002 | 5 | 5 | 4 | 3 |
|  | A1-003 | 5 | 5 | 4 | 3 |
|  | A1-004 | 4 | 4 | 3 | 3 |
|  | A1-005 | 7 | 6 | 5 | 5 |
|  | A1-006 | 4 | 4 | 2 | 2 |
|  | A1-007 | 4 | 4 | 4 | 3 |
|  | A1-008 | 5 | 4 | 4 | 4 |
|  | A1-009 | 5 | 5 | 4 | 4 |
|  | Mean±SD | 5.11±1.17 | 4.78±0.83 | 4.00±1.12 | 3.44±0.88 |
| **Examination** | A1-001 | 9 | 7 | 7 | 5 |
|  | A1-002 | 9 | 9 | 6 | 4 |
|  | A1-003 | 9 | 9 | 4 | 3 |
|  | A1-004 | 6 | 6 | 4 | 3 |
|  | A1-005 | 11 | 9 | 8 | 8 |
|  | A1-006 | 6 | 4 | 2 | 2 |
|  | A1-007 | 6 | 5 | 4 | 3 |
|  | A1-008 | 8 | 4 | 5 | 5 |
|  | A1-009 | 8 | 8 | 6 | 6 |
|  | Mean±SD | 8.00±1.73 | 6.78±2.11 | 5.11±1.83 | 4.33±1.87 |
| **Neurophysiology** | A1-001 | 7 | 8 | 8 | 8 |
|  | A1-002 | 4 | 4 | 4 | 4 |
|  | A1-003 | 4 | 4 | 4 | 4 |
|  | A1-004 | 4 | 4 | 4 | 4 |
|  | A1-005 | 6 | 6 | 6 | 6 |
|  | A1-006 | 3 | 3 | 3 | 2 |
|  | A1-007 | 5 | 5 | 4 | 4 |
|  | A1-008 | 5 | 5 | 5 | 5 |
|  | A1-009 | 4 | 4 | 4 | 4 |
|  | Mean±SD | 4.67±1.22 | 4.78±1.48 | 4.67±1.50 | 4.56±1.67 |

**Supplementary Table 4. Individual Patients data for CMTNSv2 scores and NCAM1 concentrations at baseline, Week 4 and 24**

|  | **CMTNSv2** | | | **NCAM1 (pg/mL)** | | |
| --- | --- | --- | --- | --- | --- | --- |
|  | **Baseline** | **Week 4** | **Week 24** | **Baseline** | **Week 4** | **Week 24** |
| A1-001 | 23 | 21 | 17 | 4,714.09 | 2,424.79 | 3,741.50 |
| A1-002 | 18 | 18 | 11 | 1,168.67 | 870.57 | 1,255.80 |
| A1-003 | 18 | 18 | 10 | >10,000.00 | >10,000.00 | 7,047.37 |
| A1-004 | 14 | 14 | 10 | >10,000.00 | >10,000.00 | >10,000.00 |
| A1-005 | 24 | 21 | 19 | 6,420.21 | 5,202.45 | 7,845.58 |
| A1-006 | 13 | 11 | 6 | >10,000.00 | 1,075.64 | 2,253.88 |
| A1-007 | 15 | 14 | 10 | 351.94 | 272.37 | 876.38 |
| A1-008 | 18 | 13 | 14 | 3,378.36 | 1,238.44 | 3,504.73 |
| A1-009 | 17 | 17 | 14 | 8,197.07 | 237.84 | 1,570.47 |
| Mean±SD | 17.78±3.73 | 16.33±3.54 | 12.33±4.03 | 6,025.59± 3,820.45 | 3,480.23± 3,992.43 | 4,232.86± 3,281.35 |
| Mean±SD^1)^ | 19.17±3.54 | 17.33±3.39 | 14.17±3.43 | 4.038.39± 3,022.75 | 1,707.74± 1,890.46 | 3,132.41± 2,599.02 |

^1)^ A1-003, A1-004, A1-006 were excluded from the analysis as their NCAM1 concentrations exceeded the upper limit of quantification (ULOQ; 10,000 pg/mL).

**Supplementary Table 5. Organ-specific biodistribution of CLZ-2002 in BALB/c nu/nu mice based on human Alu DNA level.**

|  | **Male** | | | | | | | **Female** | | | | | | |
| --- | --- | --- | --- | --- | --- | --- | --- | --- | --- | --- | --- | --- | --- | --- |
|  | **D1** | **D3** | **D7** | **W2** | **W4** | **W8** | **W12** | **D1** | **D3** | **D7** | **W2** | **W4** | **W8** | **W12** |
| Brain | 0/5 | 0/5 | 0/5 | 0/5 | 0/5 | 0/5 | 0/5 | 0/5 | 0/5 | 0/5 | 0/5 | 0/5 | 0/5 | 0/5 |
| Mandibular Lymphnode | 0/5 | 0/5 | 0/5 | 0/5 | 0/5 | 0/5 | 0/5 | 0/5 | 0/5 | 0/5 | 0/5 | 0/5 | 0/5 | 0/5 |
| Heart | 0/5 | 0/5 | 0/5 | 0/5 | 0/5 | 0/5 | 0/5 | 0/5 | 0/5 | 0/5 | 0/5 | 0/5 | 0/5 | 0/5 |
| Lungs | 0/5 | 0/5 | 1/5  (18.01) | 0/5 | 0/5 | 0/5 | 0/5 | 0/5 | 0/5 | 0/5 | 0/5 | 0/5 | 0/5 | 0/5 |
| Liver | 0/5 | 0/5 | 0/5 | 0/5 | 0/5 | 0/5 | 0/5 | 0/5 | 0/5 | 0/5 | 0/5 | 0/5 | 0/5 | 0/5 |
| Spleen | 0/5 | 0/5 | 0/5 | 0/5 | 0/5 | 0/5 | 0/5 | 0/5 | 0/5 | 0/5 | 0/5 | 0/5 | 0/5 | 0/5 |
| Pancreas | 0/5 | 0/5 | 0/5 | 0/5 | 0/5 | 0/5 | 0/5 | 0/5 | 1/5  (59) | 0/5 | 0/5 | 0/5 | 0/5 | 0/5 |
| Kidney | 0/5 | 0/5 | 0/5 | 0/5 | 0/5 | 0/5 | 0/5 | 0/5 | 0/5 | 0/5 | 0/5 | 0/5 | 0/5 | 0/5 |
| Mesenteric Lymph node | 0/5 | 0/5 | 0/5 | 0/5 | 0/5 | 0/5 | 0/5 | 0/5 | 0/5 | 0/5 | 0/5 | 0/5 | 0/5 | 0/5 |
| Testis | 0/5 | 0/5 | 0/5 | 0/5 | 0/5 | 0/5 | 0/5 | 0/5 | 0/5 | 0/5 | 0/5 | 0/5 | 0/5 | 0/5 |
| Femoral Muscle | 2/5  (21.93) | 1/5  (19.48) | 1/5  (173.45) | 0/5 | 0/5 | 0/5 | 0/5 | 1/5  (116.73) | 3/5  (66.87) | 0/5 | 0/5 | 0/5 | 0/5 | 0/5 |
| Gastrocnemius | 5/5  (7090.76) | 5/5  (3199.98) | 5/5  (714.25) | 5/5  (210.73) | 5/5  (129.64) | 3/5  (19.85) | 1/5  (35.45) | 5/5  (2665.69) | 5/5  (1472.61) | 5/5  (595.96) | 4/5  (45.87) | 4/5  (36.81) | 0/5 | 0/5 |
| Inguinal Lymphnode | 0/5 | 0/5 | 0/5 | 0/5 | 0/5 | 0/5 | 0/5 | 0/5 | 0/5 | 0/5 | 0/5 | 0/5 | 0/5 | 0/5 |
| Bonemarrow | 0/5 | 0/5 | 0/5 | 0/5 | 0/5 | 0/5 | 0/5 | 0/5 | 0/5 | 0/5 | 0/5 | 0/5 | 0/5 | 0/5 |
| Blood | 0/5 | 0/5 | 0/5 | 0/5 | 0/5 | 0/5 | 0/5 | 0/5 | 0/5 | 0/5 | 0/5 | 0/5 | 0/5 | 0/5 |

Detected animal number / Total animal number

Parentheses: Values represent the mean concentration of human Alu in animals where the analyte was detected (pg)

**Supplementary Table 5. materials and methods**

**Animals and Administration**

Specific-pathogen-free (SPF) BALB/c-nu/nu mice (7 weeks old) were utilized for the in vivo biodistribution assessment. All animal experiments and procedures were conducted in compliance with guidelines and approved by the institutional Animal Care and Use Communication (IACUC) of Chemon Inc. The animals were housed under standard controlled environmental conditions. The test article, CLZ-2002, was administered as a single intramuscular injection at a dose of 1.0 x 10^6^ cells per animal. To achieve this, a total volume of 200uL was equally distributed into four 50Ul injection sites across the bilateral femoral and gastrocnemius muscles using a 31-gauge syringe.

**Quantitative Real-Time PCR (qPCR) Analysis**

For the temporal biodistribution analysis, animals were euthanized at 1, 3, and 7 days, and 2, 4, 8, and 12 weeks post-injection. A total of 15 target and non-target tissues were systematically collected: brain, mandibular lymph node, heart, lungs, liver, spleen, pancreas, kidneys, mesenteric lymph node, testes/ovaries, femoral muscle, gastrocnemius, inguinal lymph node, bone marrow, and blood. To strictly prevent cross-contamination, organs anatomically farthest from the injection site were harvested first, and all dissection tools were rigorously sterilized with ethanol between individual organ isolations The presence and systemic distribution of the injected human cells within the murine tissues were quantified by targeting the human-specific Alu gene. The qPCR analysis was performed using a target-specific primer and probe set (Forward: 5'-TTA GCC GGA CGT AGT GGC-3', Reverse: 5'-GCA ATC TCG GCT CAC TGC AA-3', Probe: 5'-FAM-AGC TAC TCG GGA GGC TGA GGC AGG A-BHQ1-3')

**Results**

Following a single intramuscular administration of CLZ-2002, no mortality or test article-related severe systemic abnormalities were observed throughout the 12-week experimental period. Although transient localized swelling at the injection sites was universally noted on the day of dosing (Day 0), these reactions completely resolved by Day 1. The in vivo biodistribution profile, evaluated by measuring human Alu gene concentrations via qPCR, demonstrated that the administered cells predominantly localized and were retained at the primary injection sites. Specifically, the human Alu gene remained detectable in the femoral muscles for up to 7 days and in the gastrocnemius muscles for up to 4 weeks in both sexes, with persistence observed up to 12 weeks post-injection in male mice. In contrast, human Alu levels in non-target organs and systemic circulation—including blood and bone marrow—remained largely below the lower limit of quantification (LLOQ), with only minor, transient detections in the pancreas (female, Day 3) and lungs (male, Day 7). Conclusively, intramuscularly injected CLZ-2002 remains primarily confined to the targeted skeletal muscles without exhibiting widespread systemic distribution, supporting a favorable local safety profile.
